# Optimal Stereoacuity Reveals More Than Critical Time in Patients With Intermittent Exotropia

**DOI:** 10.3389/fnins.2020.00133

**Published:** 2020-02-18

**Authors:** Haoran Wu, Xiaoning Li, Yao Tang, Qinglin Xu, Xuhong Zhang, Lu Zhou, Weizhong Lan, Bin Zhang, Zhikuan Yang

**Affiliations:** ^1^Aier School of Ophthalmology, Central South University, Changsha, China; ^2^Aier Institute of Optometry and Vision Science, Changsha, China; ^3^Aier School of Optometry and Vision Science, Hubei University of Science and Technology, Xianning, China; ^4^College of Medicine, Zhejiang University, Hangzhou, China; ^5^Department of Ophthalmology, Children’s Hospital of Nanjing Medical University, Nanjing, China; ^6^College of Optometry, Nova Southeastern University, Davie, FL, United States

**Keywords:** intermittent exotropia, stereopsis, temporal integration, optimal stereoacuity, critical time

## Abstract

**Synopsis:**

Both optimal stereoacuity and integration time to achieve that are impaired in patients with intermittent exotropia. The deterioration of stereoacuity is more revealing since it correlates well with exotropia control score.

**Background:**

Despite the periodic misalignment of two eyes, some intermittent exotropia (IXT) patients exhibit normal stereoacuity, particularly when evaluated with static tests. It is not clear if the temporal integration process of stereopsis is altered in IXT patients, thus warranting further research.

**Methods:**

IXT patients (*n* = 29) and age-matched normal controls (*n* = 36) were recruited. Static stereopsis was measured with the Titmus stereoacuity test. In computer-generated random dots tests, stereoacuity was measured with a stimuli presentation duration varying from 100 to 1,200 ms. And the relationship between stereoacuity and stimuli duration was fitted into a quadratic model. Optimal stereoacuity was achieved when fitted curve flattened and the critical integration time was the duration needed to achieve optimal stereoacuity.

**Results:**

IXT patients were not found to differ significantly from control subjects under the Titmus test, while the Random Dots stereotest showed significantly worse optimal stereoacuity and significantly longer critical integration time. Multiple regression analysis showed that age (*R* = −4.83; *P* = 0.04) had statistically significant negative correlation on the critical integration time, age (*R* = −6.45; *P* = 0.047) and exotropia control scores (*R* = 60.71; *P* = 0.007) had statistically significant effects on optimal stereoacuity.

**Conclusion:**

The temporal integration for stereopsis is impaired in IXT patients, requiring longer critical integration time to achieve elevated optimal stereoacuity.

## Introduction

Stereopsis is the finest form of binocular visual processing, in which the image disparity between two eyes is extracted to achieve depth perception (Blake and Wilson, 2011). Stereopsis in humans is absent at birth, emerges around 3 months of age and gradually reaches adult levels around 5 years of age (Aslin, 1977; Fox et al., 1980; Birch et al., 1982; Birch et al., 1985; Giaschi et al., 2013). It is common knowledge that binocular vision adapts to natural occurring disparities, for what concerns stereopsis (Sprague et al., 2015; Gibaldi et al., 2017) and binocular coordination (Gibaldi and Banks, 2019). Due to the high level of neural plasticity in early life, abnormal binocular visual experience often can quickly disrupt this developmental process (Wiesel and Hubel, 1963; Birch et al., 1998).

Intermittent exotropia is a condition in which one or two eyes occasionally deviate outward. It accounts for the majority of exotropia reported worldwide (Govindan et al., 2005), and affects approximately 1 in 30 preschool-aged children in China (Pan et al., 2016). During misalignment, decorrelated binocular inputs impair the normal development of binocular vision. However, due the intermittent nature of exotropia, both eyes are not always misaligned. During alignment, correlated binocular inputs promote the development of binocular vision. Therefore, IXT patients show an understandably wide range of binocular deficits, from having no binocular fusion to normal stereopsis (Lee et al., 2014). Distance stereoacuity has been used clinically to evaluate binocular control, with decreasing stereoacuity indicating increased severity of IXT (Mohney and Holmes, 2006; Holmes et al., 2007; Hatt et al., 2007). Stereoacuity is also used as an index to determine the optimal time for corrective surgery (Holmes et al., 2011).

In previous studies, stereopsis was mostly measured using static stimuli, such as the Titmus test, TNO test (Mix, 2015), where subjects were allowed to view the stimulus as long as they preferred. Although vision involves the processing of both spatial and temporal information, the temporal aspect of stereopsis has been studied little in IXT patients. In normal subjects, stereoacuity improves with viewing duration and often reaches optimal levels at around 100 ms (Harwerth et al., 2003). Those with strong sensory ocular dominance tend to have longer critical integration time (Tmin) to reach similar levels of stereoacuity than those with balanced eyes (Wu et al., 2018). With mismatched binocular inputs during misalignment, it is natural to think that the Tmin would be longer in IXT patients, but this has not been tested before. The only related study reported opposite findings. Using computer-generated stimuli, (Harwerth et al., 2003) reported unchanged Tmin despite impaired stereoacuity measured with Gabor patches and random dots (RD). The study’s relatively small sample size and history of corrective surgery in strabismic subjects make IXT population inferences difficult (Harwerth et al., 2003). There was also a lack of information provided about ocular deviation and eye position control. Therefore, new studies are warranted to address this question.

The aim of this study is to investigate whether Tmin is longer in patients with IXT. If that is the case, determining whether the changes in Tmin are closely correlated with exotropia control scores or ocular deviation could support future clinical evaluation.

## Materials and Methods

### Subjects

A total of 29 IXT patients were recruited from Changsha Aier Eye Hospital (Changsha, China). The inclusion criteria were: 1) best corrected visual acuity for each eye ≥ 20/20, 2) anisometropia ≤ 1D, 3) basic type IXT, 4) possessing stereopsis indicated by synoptophore, 5) no previous surgical or non-surgical treatment for IXT other than refractive correction and 6) no history of ocular surgery or trauma. Thirty-six normal subjects, who were patients at the same hospital for refractive error examination, served as controls. The control inclusion criteria were: (1) best corrected visual acuity for each eye ≥ 20/20, (2) anisometropia ≤ 1D, (3) no strabismus, and (4) no history of ocular surgery or trauma. Written informed consent was obtained from all subjects and/or their parents (for those younger than 18 years of age) after providing an explanation of the study’s nature and possible consequences. The protocol for the study was approved by the Institutional Review Board of Aier Eye Hospital Group and followed the tenets of the Declaration of Helsinki.

Fusion, divergence and convergence were evaluated with a model synoptophore (Clement Clarke International Ltd., London, United Kingdom). Near stereoacuity was tested using a Titmus stereogram (Stereo Optical Co., Inc., Chicago, IL, United States) and the RD stereotest. For IXT patients, exotropia control was measured using the Office Control Score (Mohney and Holmes, 2006), which ranges from 0 (phoria, best control) to 5 (constant exotropia, worst control), and ocular alignment was assessed at a distance of 6 m using the prism and alternative cover test (Ansons and Davis, 2014).

### The RD Stereotest

**FIGURE 1:**
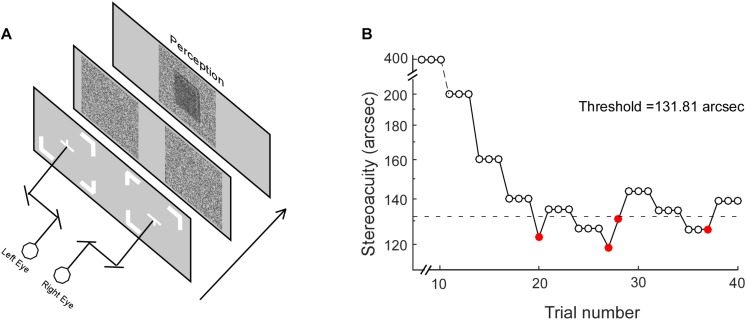
The Random Dot test. **(A)** Stereoacuity was measured with different stimulus presentation durations, including 50, 100, 200, 400, 600, 800, 1,000, and 1,200 ms. **(B)** For each stimulus presentation duration, the stereo threshold was measured with a staircase procedure. Open and closed red circles represent correct and incorrect responses, respectively. The dotted line represents the final stereo threshold averaged from the last four reversals.

The RD stimuli for testing stereopsis was programmed using commercial software (MATLAB, version 2012Rb; MathWorks, Natick, MA, United States) (Wu et al., 2018). The visual stimuli were presented on a CRT screen (Trinitron CPD-E200, 17 inches, 43.18 × 32.39 cm, 1024 × 768 resolution; 105 Hz; Sony Corporation, Tokyo, Japan), with Gamma correction for linearity, against a uniform background (50 cd/m^2^). The monitor was viewed at a distance of 115 cm. Each eye viewed a pattern of RD subtended 3.3° × 3.3°. Using a double stereoscope, the subjects’ left and right eyes viewed fixation marks (Figure 1A) in the center of each half of the screen, where the half images of the RD would be presented. The fixation marks were an upward-pointing and downward-pointing “T” for the left and right eyes, respectively. Subjects adjusted the mirrors to fuse the two symbols, and were allowed to begin their trial only when they saw a complete square box with a cross in the center. For each trial, the subjects were instructed to determine whether the central square (1° × 1°) was standing in front of the background or falling behind the background. For a fixed presentation duration, the amount of disparity varied for each trial and the disparity threshold was measured using a 3-down 1-up staircase algorithm (Figure 1B). The initial stimulus values were 400 aresec. The decreasing/increasing rate was 30% before the first reversal, and 15% afterward. Each staircase ended when it reached six reversals and the values from the last four reversals were averaged as the final stereo threshold.

**FIGURE 2:**
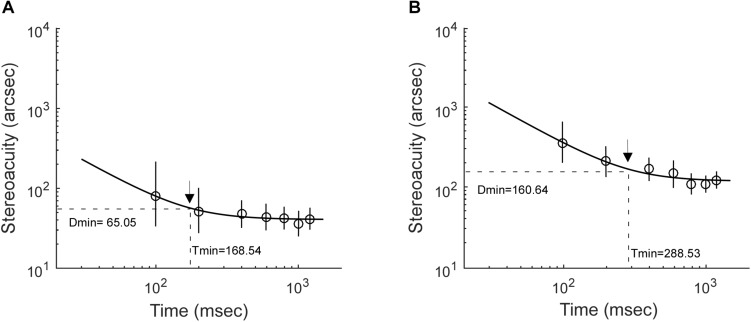
Examples illustrating how stereoacuities change with stimulus presentation durations. **(A)** A control subject. **(B)** An IXT patient. The black arrow indicates the location of optimal stereoacuity (Dmin) and the critical integration time (Tmin) required to achieve it.

Stereoacuity was measured with different RD presentation durations, including 100, 200, 400, 600, 800, 1,000, and 1,200 ms. It is worth noting that on a stimulus presentation duration of 100 ms, the display in the current study with a frequency of 105 Hz would provide a mismatch of up to ±4.8 ms. The stereo thresholds (*th*) versus viewing durations were fitted into a quadratic model of *th* = *h0 ^∗^ sqrt(t^–2^* + *Tmin^–2^*), where *th* was the stereoacuity at a given presentation duration (*t*), *h0* determined the vertical height of the function, *Tmin* was the critical time constant at which the stereoacuity no longer changes (the optimal stereoacuity, Dmin). Figure 2 shows the stereo threshold as a function of viewing duration for a normal subject (Figure 2A) and an IXT patient (Figure 2B).

### Data Analyses and Statistics

Statistical analyses were performed using the R programming package (version 3.2.2^1^; The R Foundation, Vienna, Austria). The Shapiro–Wilk test was used to test the normality of data. The data of each subject’s age, refraction, fusion, divergence, convergence and ocular alignment followed normal distribution; therefore, a mean and standard deviation were used for description and a *T*-test was used for comparison. The data of each subject’s Titmus stereoacuity and exotropia control score did not follow normal distribution; therefore, a median and range were used for description and a Kolmogorov–Smirnov test was used for comparison. A chi-square test was used to compare gender difference. Multiple parameter linear regression was used to explore the correlation between Tmin/Dmin and several possible parameters. Differences of *p* < 0.05 were defined as statistically significant.

## Results

Twenty-nine IXT patients (14 males, 48.28%) and thirty-six control subjects (17 males, 47.22%) were recruited during the period of August 2018 to April 2019. There was no significant difference in gender, age, refraction, fusion, divergence or convergence between the IXT and control groups (Table 1).

**TABLE 1:** Patient characteristics in control subjects and IXT patients.

**Characteristic**	**Control**	**IXT**	**Statistic**	***p*-**
	**subjects**	**patients**	**value**	**value**
	**(*n* = 36)**	**(*n* = 29)**		
Gender (Male:Female)	17:19	14:15	<0.01	0.57^a^
Age (Years old)	16.78 ± 5.73	16.41 ± 6.13	0.25	0.81^b^
Refractive error (Diopter)	−2.86 ± 2.30	−1.87 ± 2.40	–1.69	0.10^b^
Fusion (Prism diopter)	29.56 ± 8.89	27.66 ± 8.19	0.89	0.38^b^
Divergence (Prism diopter)	−7.28 ± 1.95	−6.93 ± 3.35	–0.52	0.60^b^
Convergence (Prism diopter)	22.28 ± 8.51	20.72 ± 8.04	0.75	0.46^b^
Ocular alignment (Prism diopter)	–	−36.38 ± 19.27	–	–
Exotropia control score	–	2(1–4)	–	–

*^*a*^χ^2^ test. ^*b*^*t*-test.*

**FIGURE 3:**
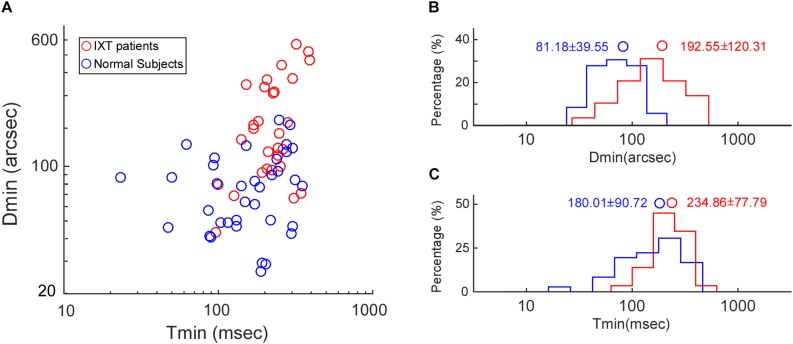
Population data on Dmin and Tmin. **(A)** Scatter plot showing the relationship between Tmin and Dmin in the control subjects (blue dots) and the IXT patients (red dots). **(B)** Histogram showing the distribution of Dmin in the control subjects (blue bars) and the IXT patients (red bars). **(C)** Histogram showing the distribution of Tmin in the control subjects (blue bars) and the IXT patients (red bars). Circles represent the mean values.

For the Titmus test, IXT patients (median: 80 arcsec, range: 20–320 arcsec) did not differ significantly from control subjects (median: 80 arcsec, range: 20–160 arcsec, KSSTAT = 0.17, *p* = 0.71). For the RD test, the relationship between Tmin and Dmin in the control subjects and IXT patients has been plotted in Figure 3A. Dmin for IXT patients was 192.55 ± 120.31 arcsec, significantly worse than the control group (81.18 ± 39.55 arcsec, *t* = −5.22, *p* < 0.01; see Figure 3B). Tmin for the IXT group was 234.86 ± 77.79 ms, significantly longer than the normal group (180.01 ± 90.72 ms, *t* = −2.58, *p* = 0.01; see Figure 3C).

**FIGURE 4:**
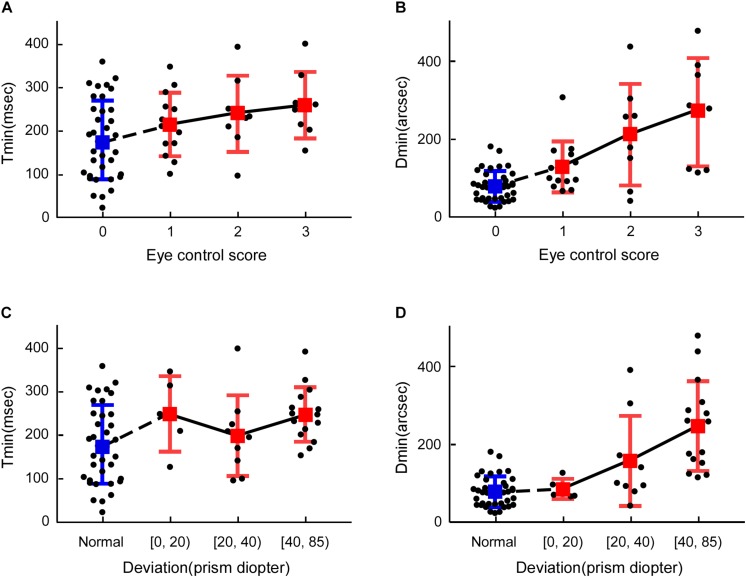
Comparisons between Dmin, Tmin, exotropia control score and ocular deviation. **(A)** Tmin vs. exotropia control score, **(B)** Dmin vs. exotropia control score, **(C)** Tmin vs. ocular alignment, **(D)** Dmin vs. ocular alignment. The blue marker represents the control subjects, and the red marker represents the IXT patients.

To further explore how Tmin and Dmin were affected by IXT characteristics, the relationship between Tmin and Dmin, ocular alignment and exotropia control score has been summarized in Figure 4. Multiple regression analysis showed that age (*R* = −4.83; *P* = 0.04) had statistically significant negative correlation on Tmin, age (*R* = −6.45; *P* = 0.047) and exotropia control scores (*R* = 60.71; *P* = 0.007) had statistically significant correlation on Dmin.

## Discussion

In this study, we found that Dmin is more than doubled in IXT subjects, while Tmin only increases by roughly 30%. The age had statistically significant negative correlation on Tmin and Dmin. Moreover, Dmin is closely associated with exotropia control scores while Tmin is not.

### Comparison to Existing Studies

The finding of increased Dmin in our study agreed with many previous studies reporting that stereoacuity is impaired in persons who have abnormal visual experience early in life (Hatt et al., 2007; Zhou et al., 2019). However, the increased Tmin does not agree with early studies. According to Harwerth et al. (2003), Tmin remained constant despite elevated Dmin. This disagreement may be due to several reasons. In the previous study, 12 monkey subjects and two human subjects with microstrabismus were examined. Among the monkey subjects, nine experienced alternating defocus to induce strabismus from 3 weeks to 9 months of age, and three had surgically induced esotropia for an unknown period of time (Harwerth et al., 2003). In our study, all 29 IXT subjects had not received corrective surgeries. The natures of the abnormal binocular experience also differ. During the period of alternating defocus rearing, the eyes never receive clear images simultaneously, although the eyes might still be aligned. For IXT patients, images from both eyes are clear, but mismatched. The two human subjects included in Harwerth’s study were diagnosed with microstrabismus, making a direct comparison to results from IXT patients difficult.

Previous studies have proved that stereopsis emerges around 3 months of age and gradually reaches adult levels around 5 years of age in normal visual development (Aslin, 1977; Fox et al., 1980; Birch et al., 1982; Birch et al., 1985; Giaschi et al., 2013). In the current study, all the IXT patients were more than 5 years old, however, negative correlation of age were still found on Tmin/Dmin. The onset age of IXT is usually from one to 4 years old (Clarke et al., 2007; Buck et al., 2009),we speculate that perhaps the intermittent decorrelated binocular inputs delays and prolongs the development of stereopsis. To clarify the specific mechanism, it may be necessary to analyze the onset age, duration and severity of IXT. Since these characteristics of IXT are often vague, so we may need to conduct further research in the future.

### Stereopsis and Development

Three-dimensional depth perception relies in part on the binocular fusion of horizontally disparate stimuli presented to the left and right eye, visual disparity is encoded in the cortex. Binocular neurons in V1 of awake monkeys are selective for absolute, not relative, disparity (Cumming and Parker, 1999). Higher visual areas, such as V2, V3, V4, and MT, are more engaged with stereoscopic processing than the primary visual cortex (Skrandies, 2001).

Compared with psychophysical methods, visual evoked potentials (VEP) based on cortical neuron electrophysiological records can more objectively reflect the process of stereo information processing. Based on dynamic random dots stereograms (dRDS), studies show that the stereoscopic VEP activity amplitude of patients with impaired binocular integration decreases significantly (Wesemann et al., 1987; Skrandies, 1995). There is a strong correlation between electrophysiological changes and perceptual impairment measured by psychophysical methods (Skrandies, 2009). In addition, previous studies have also found that binocular VEP summation in stereo deficient adults is much lower than normal adults (Shea et al., 1987).

The visual impairments are often caused by abnormal visual input during the early stages of visual system development (Hadad et al., 2015). Amblyopia is the most common disorder of spatial visual development, which often associated with the presence of strabismus, refractive errors, or form deprivation early in life. Amblyopes suffer not only from sensory deficits, but also from deficits not simply explained by low-level considerations, like second-order processing, contour integration, temporal, spatial and/or capacity limits of attention, and motion (Levi et al., 2015). Although IXT patients have normal visual acuity, intermittent abnormal visual input may also cause similar visual impairments, further studies are warranted.

### Dmin, Tmin, and Ocular Control

Dmin correlated well with exotropia control score. Worse exotropia control scores indicate that eyes are more frequently misaligned and have shorter periods of exposure to correlated binocular images (Mohney and Holmes, 2006), larger deviations caused large shifts in monocular images during the misaligned phase, creating greater disconcordance between binocular signals, which led to a greater loss of binocular neurons (Smith et al., 1997).

Tmin did not correlate well with ocular control and ocular alignment. At one hand, the imprecise control of vergence position in IXT patients could lead to longer Tmin. When the convergence was carefully controlled, reliable stereoscopic form recognition in random-dot stereograms has been demonstrated for very brief stimulus exposure times (1 ms) (Uttal et al., 1994). Another proof is that, in strabismic amblyopic monkeys, the response latency of V1 neurons dominated by an amblyopic eye is even shorter than that of neurons dominated by a non-amblyopic eye (Bi et al., 2011; Wang et al., 2017). Similarly, the response latency measured with multifocal visually evoked potentials is shorter for an amblyopic eye when compared to a fellow eye. In both studies, the response latencies were measured monocularly with the effect of imprecise control of vergence removed (Greenstein et al., 2008). On the other hand, Tmin is shorter in humans with balanced eyes and significantly longer in subjects with strong ocular dominance (Wu et al., 2018). Therefore, it is not the time need to for information to reach binocular cells, rather the time needed for binocular cells to integrate the signals from two eyes and to extract stereopsis that causes an longer Tmin. It is possible that the variance in ocular dominance in our subjects might have masked the correlation between Tmin and ocular deviation and eye control (Wu et al., 2018). In future studies, we plan to quantify the effect of ocular control, ocular deviation and sensory dominance on IXT patients.

### Clinical Applications

Although our study reported significantly longer Tmin values, the relative increment was only 30%. A 50 ms difference would hardly be noticed in clinic, since most clinical stereopsis testing used allow patients sufficient time, usually several seconds, to view printed stimuli. That might explain why so few previous studies addressed the temporal aspect of the stereoacuity test. In the past, most of the training programming have been focusing on how to improve Dmin. It is well established that stereoacuity can be improved after a 3D movie viewing experience (Bridgeman, 2014), and that 3D video game play can improve stereopsis (Li et al., 2018). It is not clear whether temporal integration time is shortened after visual training as those new areas remain unexplored.

With more clinics equipped with electronic visual function test units, precise controlling of the stimulus presentation duration has become practical. It would provide great value to apply both Dmin and Tmin to binocular research. For example, Dmin has long been used as sensitivity index to quantify the ocular control and progression of IXT (Mohney and Holmes, 2006; Hatt et al., 2007; Holmes et al., 2007). The deterioration of stereoacuity usually indicates a necessity for corrective surgery (Uttal et al., 1994). However, stereoacuity repeatability in IXT is quite low, even for measurements taken during the same day (Hatt, 2008). It is not clear if longer Tmin contributes to decreased stability.

## Data Availability Statement

The datasets generated for this study are available on request to the corresponding author.

## Ethics Statement

The studies involving human participants were reviewed and approved by the Institutional Review Board of Aier Eye Hospital Group. Written informed consent to participate in this study was provided by the participants’ legal guardian/next of kin.

## Author Contributions

HW, XL, QX, XZ, LZ, WL, BZ, and ZY conceived and designed the experiments. HW, YT, and QX performed the experiments. HW, XL, YT, QX, XZ, and LZ analyzed the data. XL, WL, BZ, and ZY contributed reagents, materials, and analysis tools. HW, XL, QX, XZ, LZ, BZ, and ZY wrote the manuscript.

## Conflict of Interest

The authors declare that the research was conducted in the absence of any commercial or financial relationships that could be construed as a potential conflict of interest.

## Footnotes

1 http://www.R-project.org
